# Case Report: A novel approach to pseudoportomesenteric hypertension with refractory chylothorax and ascites

**DOI:** 10.3389/fgstr.2025.1499385

**Published:** 2025-10-27

**Authors:** Ahmet Sakiri, Annalise De Marco, Shreekar Patel, Naser Khan, Thayer Hamoudah, Altaf Dawood, Rajesh Kakarla

**Affiliations:** Department of Gastroenterology, Mercyhealth, Rockford, IL, United States

**Keywords:** pseudoportomesenteric hypertension, refractory ascites, SMV stent, pancreatic neuroendocrine tumor, chylothorax

## Abstract

Pseudoportomesenteric hypertension (PPMH) is a rare form of non-cirrhotic portal hypertension caused by increased pressure in the portal and mesenteric venous systems. Unlike traditional portal hypertension, which arises from liver cirrhosis, PPMH stems from conditions that are extrahepatic in etiology, such as venous thrombosis, external compression by intra-abdominal masses, or congenital anomalies. Treatment is aimed at addressing the underlying cause of obstruction. We report a case of PPMH secondary to a pancreatic neuroendocrine tumor (PNET). A female in her 60s presented with a six-month history of diarrhea, 30-pound weight loss, and dyspnea. Initial evaluation revealed massive right pleural effusion with subtotal lung collapse, cardio-mediastinal shift, and sub-segmental pulmonary emboli. Additionally, she had extensive four-quadrant ascites and a heterogeneous mass at the pancreatic head and uncinate process encasing the superior mesenteric artery (SMA), with mass effect on the superior mesenteric vein (SMV). The patient developed refractory ascites and chylothorax, which were managed with a chest tube and abdominal drain placement. Retroperitoneal lymph node biopsy confirmed a well-differentiated grade 2 primary PNET. Following a multidisciplinary assessment, the patient underwent stent placement in the SMV. Initial trans-hepatic portal venography showed a portal vein pressure of 12 cm H2O and a significantly elevated SMV pressure of 32 cm H2O, indicating severe obstruction at the portal-mesenteric confluence. Extensive mesenteric collateralization and large duodenal and mesenteric varices were noted. Angioplasty and stenting of the SMV were performed, resulting in a 10 cm H2O reduction in the pressure gradient, improved antegrade hepatopetal flow, and resolution of varices. This case highlights the importance of recognizing extrahepatic causes of portal hypertension, particularly in patients with malignant tumors, and the role of palliative endovascular interventions in managing the sequelae of PPMH.

## Introduction

Pseudoportomesenteric hypertension (PPMH) is a rare form of non-cirrhotic portal hypertension caused by extrahepatic compression or obstruction of the mesenteric or portal venous system. When the superior mesenteric vein (SMV) or portal vein is compressed by tumor or lymphadenopathy, patients can develop refractory ascites, variceal formation, and chylous effusions. Pancreatic masses, including pancreatic neuroendocrine tumors (pNETs), can lead to compression and significantly elevate portal pressures (1). We present a case of unresectable pNET causing SMV obstruction and subsequent pseudoportomesenteric hypertension, resulting in massive ascites and chylothorax. We highlight the successful use of an SMV stent to palliate refractory fluid collections and improve the patient’s quality of life.

## Case report

A woman in her 60s, with a known carrier status for cystic fibrosis and newly diagnosed exocrine pancreatic insufficiency, presented to the emergency department with a six-month history of loose frothy bowel movements, progressive fatigue, exertional dyspnea, and a 30-pound unintentional weight loss. She reported frequent small-volume bowel movements with associated urgency, tenesmus, hypogastric cramping, and occasional streaks of blood on wiping. Additionally, she noted early satiety, post-prandial bloating, and abdominal distension.

**Figure 1:**
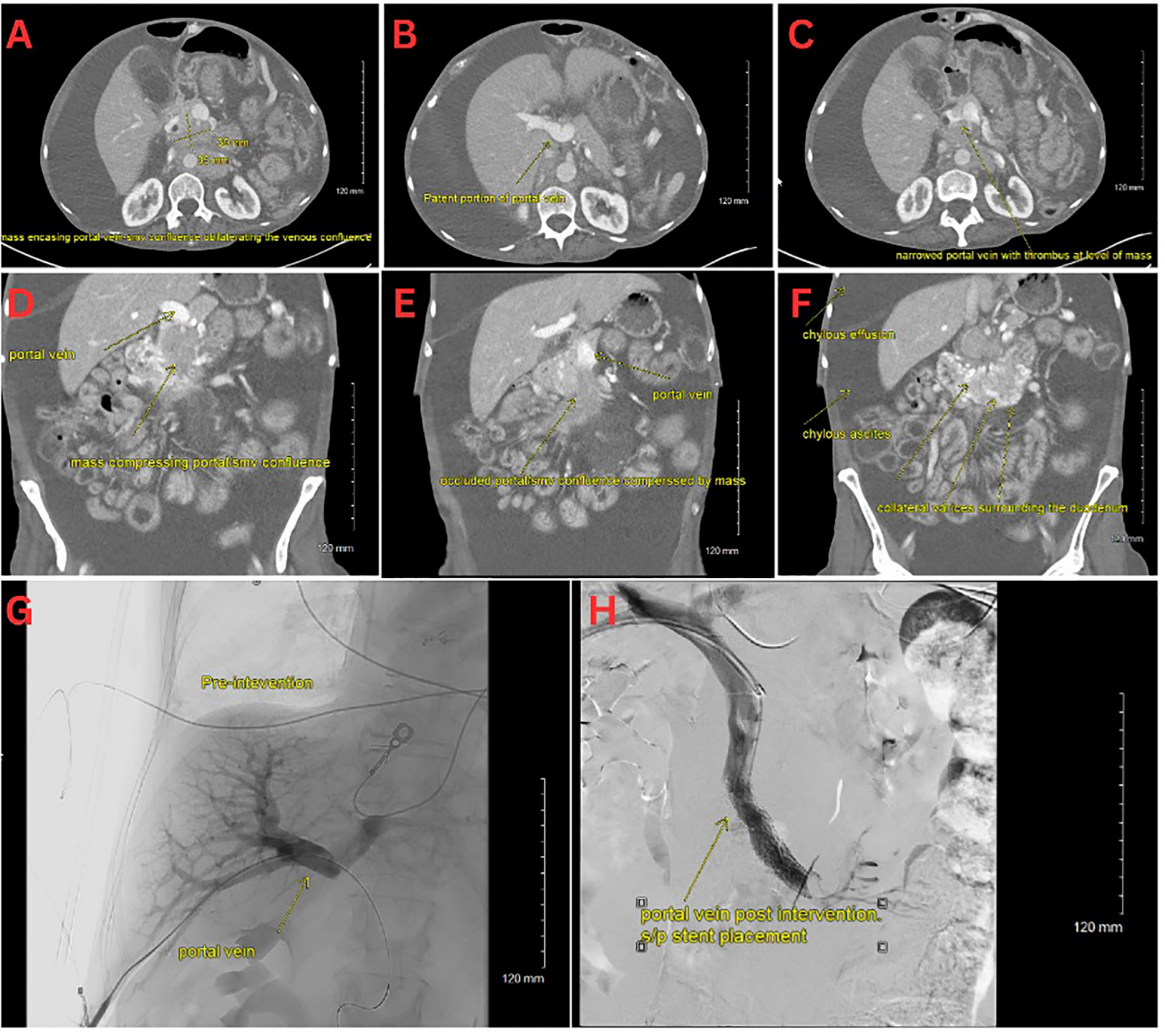
**(A–C)** CT scan with an axial view of the pNET mass. **(D–F)** CT scan with a coronal view of the pNET mass. **(G)** Venogram showing portal vein prior to stent deployment with no distal contrast visualized. **(H)** Venogram post SMV stent deployment with subsequent patency.

On initial evaluation, she appeared cachectic, tachycardic (110 bpm), tachypneic, and had an oxygen saturation of 87% on room air. She had conjunctival pallor, severely diminished breath sounds on the right side, massive abdominal distension with diffuse dullness to percussion, and bilateral pitting edema extending to the thighs. Notable laboratory results included mild thrombocytosis (platelets 471 x 10^3/µL), hypokalemia (K 2.7 mmol/L), hypoalbuminemia (3.2 g/dL), and hypomagnesemia (0.9 mg/dL), and an elevated troponin levels (28, 27, 24 ng/L). A chest CT showed a massive right pleural effusion with leftward cardio-mediastinal shift and near-complete right lung collapse. Additionally, segmental pulmonary emboli were found in the left lower lobe (**Figures 1A–F**). Abdominal CT revealed a heterogeneous pancreatic head/uncinate mass with encasement of the superior mesenteric artery (SMA) and mass effect on the SMV, retroperitoneal lymphadenopathy, and sclerotic bone lesions.

Due to marked ascites, a paracentesis was performed that demonstrated a serum-ascites albumin gradient (SAAG) of 2.3, suggesting portal hypertension. Cytology was negative for malignant cells. After chest tube placement, the pleural fluid eventually became milky, with triglyceride levels of 343 mg/dL, confirming chylothorax. She required frequent large-volume paracenteses and chest tube drainage for refractory ascites and chylothorax.

**Figure 2:**
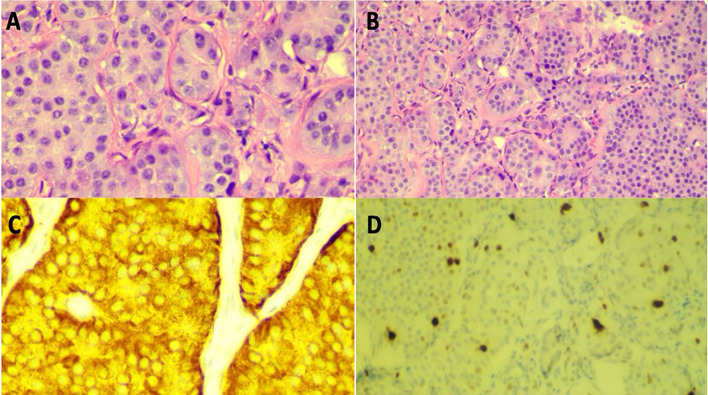
**(A)** High power (20x) view of H&E stain of retroperitoneal lymph node biopsy. **(B)** Low power (10x) view of H&E stain of retroperitoneal lymph node biopsy. **(C)** High power (20x) view Synaptophysin stain of lymph node biopsy in high power (20x) view. **(D)** Ki-67 positive stain (~4% positive), indicating well-differentiated grade 2 neuroendocrine tumor in low power (10x) view.

Biopsy options for the pancreatic mass were limited by large duodenal varices. Thus, a CT-guided retroperitoneal lymph node biopsy was performed, confirming a well-differentiated grade 2 pancreatic neuroendocrine tumor (**Figure 2**). Ultrasound duplex of the abdomen and pelvis was conducted to assess mesenteric vessel flow. This revealed significant hemodynamic stenosis exceeding 70% at the distal superior mesenteric artery, consistent with prior cross-sectional imaging demonstrating narrowing and encasement by a mass in the pancreatic head and uncinate process. Additionally, marked splanchnic hypertension was noted, with evidence of hemodynamically significant obstruction at the portal-mesenteric confluence.

Interventional radiology performed a transhepatic portal venogram, demonstrating a 20 cm H2O gradient across the SMV obstruction, along with extensive varices. A 12 mm x 60 mm self-expanding stent was deployed, reducing the gradient from 20 to 10 cm H2O and restoring antegrade hepatopetal flow (**Figures 1G–H**). Post-procedure, her abdominal distension, and chylothorax gradually improved, and large-volume paracenteses were no longer required. She was started on total parenteral nutrition (TPN) and long-acting octreotide for symptom management.

A Ga68-DOTATATE PET/CT confirmed SSTR2-positive pancreatic head mass, with hepatic and osseous metastases. She was started on capecitabine and temozolomide chemotherapy, but tolerability issues led to early discontinuation. She continued octreotide with plans for routine imaging follow-up.

## Discussion

Unlike cirrhosis, where increased intrahepatic resistance drives portal hypertension, pseudoportomesenteric hypertension constitutes a non-cirrhotic, prehepatic phenomenon arising from external compression of the portal or mesenteric venous system (2). Recognizing this distinction is crucial, as correcting the anatomical obstruction can alleviate symptoms and improve clinical outcomes. The superior mesenteric vein (SMV) is a critical component of the portal venous system, responsible for draining blood from the small intestine, cecum, and ascending colon and delivering nutrient-rich blood to the liver via its confluence with the splenic vein to form the portal vein. Any pathology that significantly narrows or occludes the SMV—such as tumor encasement—can precipitate a severe increase in splanchnic venous pressures.

In the presented case, a pancreatic neuroendocrine tumor encased the SMV, leading to significant luminal narrowing and obstruction of blood flow. This obstruction resulted in elevated splanchnic venous pressures, manifesting as refractory ascites and chylothorax due to increased hydrostatic pressure and subsequent fluid transudation into the peritoneal and pleural cavities. Symptoms can be refractory to medical therapy alone and require interventional relief of the obstruction.

Endovascular stenting offers a key palliative strategy for malignant SMV obstruction. By deploying a stent across the stenotic segment of the SMV, normal antegrade hepatopetal flow is reestablished, effectively reducing splanchnic hypertension. Maintaining SMV patency lowers the portomesenteric gradient, thereby diminishing fluid extravasation into the peritoneal and pleural spaces, allowing for the resolution of ascites and chylothorax. Additionally, restoring normal hemodynamics improved the patient’s quality of life by reducing abdominal distension, facilitating better oral intake, and decreasing reliance on paracentesis and total parenteral nutrition.

The successful outcome aligns with existing literature, which reports high technical success rates and significant symptom palliation in patients undergoing portal vein stenting for malignant obstructions. For instance, a single‐center, retrospective study by Shah et al. evaluated percutaneous transhepatic portal vein stenting (PVS) in 38 patients with pancreaticobiliary cancer and extrahepatic portomesenteric venous stenosis leading to refractory ascites or variceal bleeding (3). In the 28 patients treated primarily for these complications, PVS achieved a 93% technical success rate and sustained symptomatic relief for an average of 87% of the remaining patients. Stent patency correlated strongly with decreased ascites and stable liver volume, while no major or minor complications were observed (3). These findings underscore the potential of PVS to significantly improve the quality of life in this challenging clinical setting.

While surgical resection is ideal in localized disease, metastatic and vascularly encased tumors often preclude curative resection (4). Portal-SMV stenting is a low-risk procedure with a high clinical success rate that can provide a bridge to systemic therapies (e.g., octreotide, chemotherapy) and enhance quality of life (3). Somatostatin analogs like octreotide can slow tumor progression and reduce secretory outputs, while capecitabine–temozolomide may offer antiproliferative effects in well-differentiated pNETs (5). However, therapy must be individualized based on tolerance and patient goals.

This case underscores the importance of understanding the anatomical and physiological underpinnings of the portal venous system. It highlights the efficacy of SMV stenting as a therapeutic option for managing complications arising from malignant portal vein obstructions. Multidisciplinary care, incorporating interventional radiology, oncology, and gastroenterology, is essential in managing such cases to balance endovascular, medical, and oncological therapies. SMV stenting serves as a viable and effective intervention for patients with pseudoportomesenteric hypertension secondary to malignant obstruction, especially when surgical resection is not feasible, offering substantial improvements in clinical symptoms and overall quality of life. Future research should focus on identifying criteria for patient selection and timing of stent placement, as well as factors that may affect stent patency.

## Data Availability

The original contributions presented in the study are included in the article/supplementary material. Further inquiries can be directed to the corresponding author.
